# Miltefosine inhibits Chikungunya virus replication in human primary dermal fibroblasts

**DOI:** 10.12688/f1000research.13242.1

**Published:** 2018-01-04

**Authors:** Anuj Sharma, Manish Bhomia, Tze-Jou Yeh, Jay Singh, Radha K. Maheshwari

**Affiliations:** 1 Department of Pathology, Uniformed Services University of the Health Sciences, Bethesda, MD, 20814, USA

**Keywords:** Chikungunya virus, Akt-activation, Pi3-akt signaling inhibitors, FDA approved drug, miltefosine

## Abstract

**Background**: Chikungunya virus (CHIKV) is a re-emerging pathogen that has caused widespread outbreaks affecting millions of people around the globe. Currently, there is no specific therapeutic drug against CHIKV, with symptomatic treatment only to manage the disease. Pi3-akt signaling has been implicated in infection of several viruses including that of CHIKV. Effect of Pi3-akt signaling inhibitors on CHIKV replication was evaluated in this study.

**Methods**: Human primary dermal fibroblast cells were treated with inhibitors of the Pi3-akt signaling pathway. Suppression of CHIKV replication was evaluated as reduction in virus titer in cell supernatants. Effect of miltefosine (MF) on CHIKV replication was evaluated in pre and post treatment regimen. Inhibition of virus replication was determined by cell growth, virus titer and western blot.

**Results**: Inhibition of Akt-phosphorylation significantly inhibited CHIKV replication. No effect on CHIKV replication was observed after treatment with Pi3-kinase and mTOR activation inhibitors. Further, MF, an FDA-approved Akt-inhibitor, inhibited CHIKV replication in pre- and post-infection treatment regimens.

**Conclusion**: Data suggests that Akt-phosphorylation can be an amenable target of therapy against CHIKV infection. This is the first study to show inhibition of CHIKV replication by MF, and presents a case for further development of MF as an anti-CHIKV drug.

## Introduction

Chikungunya virus (CHIKV) is an Old World alphavirus which has caused widespread outbreaks in tropical countries around the globe ^1– 4^. Lack of herd immunity combined with increased travel across the globe, and adaptation to *Aedes albopictus* have been suggested to contribute to the world wide spread of CHIKV ^5, 6^. The population in continental United States lack immunity to CHIKV and is at high risk of CHIKV outbreak ^7^. CHIKV causes self-resolving febrile illness accompanied by arthralgia. Some studies have suggested that persistence of CHIKV in the joint tissue may be responsible for the long lasting arthralgia observed in the patients recovering from primary CHIKV infection ^8– 10^. There is no approved vaccine or specific antiviral drug to treat CHIKV infection. In our earlier study, Pi3-akt signaling was identified as one of the main pathways modulated by CHIKV infection ^11^. Others have also shown modulation of Pi3-Akt signaling by CHIKV infection ^12– 15^. Therefore, in this study, the effect of inhibition of Pi3-Akt signaling on CHIKV replication was evaluated in human primary dermal fibroblast (hPDF) cells. HPDF cells were used in this study as dermal fibroblast are one of the primary targets of CHIKV infection ^16^. Specific inhibitors of Akt-phosphorylation inhibited CHIKV replication in cell culture, suggesting Akt-phosphorylation to be important for CHIKV replication. Data mining for an FDA approved Akt-phosphorylation inhibitor, identified miltefosine (MF), which is used for treating visceral leishmaniasis ^17^. A significant inhibition of CHIKV replication was observed in the hPDF cells treated with MF before and after the infection. This inhibition of the CHIKV replication was associated with the inhibition of Akt-phosphorylation. This is the first study to report anti-CHIKV activity of MF.

## Material and methods

*Virus*: CHIKV181/25 strain of CHIKV was used in the present study and has been described earlier ^11^. Virus was a kind gift from Dr. Michael D Parker, USAMRIID, Fredrick, MD. TC83 strain of Venezuelan equine encephalitis virus (VEEV) was used. Viruses were grown in Vero cells and sucrose gradient purified before use.

*Inhibitors*: LY294002 (Catalog # 440202), Akt inhibitor IV (Akt-IV; Catalog # 124011), Akt inhibitor VIII (Akt-VIII; Catalog # 124018), Rapamycin (Catalog # 553210), H89 (Catalog # 371963), and Miltefosine (Catalog # 475841) were purchased from EMD-Millipore (Billerica, Massachusetts 01821) and solutions were made in DMSO (catalog # KP31817) as per manufacturer’s recommendation.

*Antibodies*: Following antibodies were purchased from Cell Signaling Technology, Inc., Danvers, MA: Akt pan (cat# 4691), and p-Akt (cat # 4060). Following secondary antibodies were used: Goat anti-mouse IgG HRP conjugated (Bio-rad, cat# 170-6517), and goat anti-rabbit IgG HRP conjugated (Bio-rad, cat# 170-6515). Anti-CHIKV monoclonal antibody (CHK-48) was a kind gift from Dr. Michael S. Diamond, Washington University School of Medicine. St. Louis, MO and was acquired under a material transfer agreement.

*Cells*: Human primary dermal fibroblast (hPDF) cells (Catalog # C-013-5C), growth media and supplements were purchased from Life Technologies (Grand Island, NY 14072). Cells were grown as per manufacture’s recommendation in culture media 106 (Catalog# M-106-500) supplemented with Low Serum Growth Supplement kit (Catalog# S-003-K).

*Growth curve of CHIKV181/25 in hPDF cells:* HPDF cells were plated to 80% confluence in 12 well plate. After overnight incubation, cells were infected with CHIKV181/25 at a multiplicity of infection (MOI) of 1. Cells were incubated with virus suspension for 1hr at 37°C/5% CO _2_. Unabsorbed virus was removed by two washes of cells with fresh media. Finally, 2 ml of fresh media was added to each well and cells were incubated at 37°C/5% CO _2_. Cell supernatants were collected at 6h, 12h, 24 h, 48 h and 72 h post infection from separate wells and virus titers were determined as 50% tissue culture infectivity dose (TCID50/ml).

*Treatment of cells with inhibitors and infection*: HPDF cells were grown to 75–80% confluence and treated with various inhibitors for 6 hours. Cells were then infected with CHIKV181/25 at an MOI=1 or 0.1 and incubated at 37°C/5%CO _2_ for 1 hr. Unabsorbed virus was washed by rinsing cells once with fresh culture media. Cells were then incubated in fresh media containing the respective dose of inhibitors.

*Toxicity of MF in hPDF cells:* MF is a mitotic inhibitor and therefore, effect of MF on hPDF cell proliferation was tested. HPDF cells were treated with increasing doses of MF and cell growth was assesses using MTT assay. As MF was dissolved in DMSO, the concentration used for DMSO only samples was the same as the concentration of DMSO in 40 µM MF. ****

*MF pre-treatment study*: Cells were treated with respective doses of MF for 4–6 h and then infected with CHIKV181/25 at an MOI=1 or 0.1. Unabsorbed virus was removed after 1hr and cells were rinsed once with fresh media. Cells were then incubated in fresh media containing respective doses of MF for 24 h post infection. DMSO and saline treated cells were used as controls. Experiment was performed in 12-well plates and drug treatment was done in triplicate wells. For protein assays, experiments were performed in 6-well plates and drug treatment was performed in triplicates. All experiments were repeated for reproducibility. A similar experimental set up was used to evaluate effect of pre-treatment of hPDF cells with MF on TC-83 replication.

*MF post-treatment study*: Cells were first infected with CHIKV 181/25 at an MOI=1 or 0.1. Unabsorbed virus was removed and cells were incubated in fresh media without MF, except in the group where MF treatment was done at the time of infection (ATI). Cell supernatants were replaced with fresh media containing MF at pre-determined time points of ATI, 90 min, 6 h, 12 h, or 24 h post infection. Virus titers were determined in cell supernatants 24 h post-infection. Experiments were performed in 12-well plates and drug treatment was done in triplicate wells. For TC-83 study, MF treatment at only one time point of 90 min post infection was evaluated. Experiment was repeated for reproducibility.

*Virus titer evaluation*: After 24 h post infection, cell supernatants were collected and virus titers were determine as TCID _50_/ml using Reed and Muench method as described before ^18, 19^. Briefly, 3000 cells were plated in each well of 96-well plate and incubated overnight at 37°C/5%CO _2_. Samples were serially diluted from 10 ^-1^ to 10 ^-10^ in fresh cell culture medium. 100 µl of diluted virus was added in each well, such that 8 replicates for each serial dilution were made. Plates were incubated for 3 days and cytopathic effect in cells was evaluated under a microscope. Each well was scored + or –, respectively, for the presence or absence of CPE. Cumulative percent mortality for each dilution was calculated and TCID50/ml was calculated using the following formula: 10 x [10 ^( *X*)^ x 10 ^(dilution at which percent mortality >50)^]. Where *X* = [(percent mortality>50 – 50)/(percent mortality>50 – percent mortality <50)].

*Protein analysis*: Cells were lysed with RIPA buffer (Cat# 786-489, G Biosciences, St. Louis, MO) containing phosphatase and protease inhibitors (Cat# 04693159001 and 04906845001 respectively, Roche). Samples from triplicate well for each group were pooled, and protein was estimated by Pierce BCA kits (cat# 23227, Life technologies, Grand Island, NY) as per manufacture’s protocol. Equal amount of protein was loaded on the gel (Precise 4–20% Tris-Glycine Gel, Cat# 25249, Life Technologies, Grand Island, NY). After electrophoresis, proteins were transferred on to the nitrocellulose membrane (Cat# RPN78D, GE Healthcare Life Sciences, Pittsburgh, PA). Blocking was performed using 5% non-fat dried milk in 1XTBST, and membrane was incubated with respective primary antibody at 4°C overnight over a continuous shaker. Depending on the species of the primary antibody, bands were probed either by HRP-conjugated goat anti-mouse or HRP-conjugated goat anti-rabbit antibody.

*Assessment of cell growth in CHIKV-infected and MF treated cells*: In both the pre- and post-treated study with MF, hPDF cells in 12-well plates were left with drug and virus containing supernatant for 72 h post infection. Cell supernatants were then removed and cells were washed twice with fresh culture media. Cells were then incubated at 37°C/5% CO _2_ for 4 days in fresh culture media. Cells were then fixed with 0.1% crystal violet in 10% neutral buffered formalin for 90 min. Plates were washed gently under a continuous flowing tap water for 3 min. Cells were then observed under a microscope for growth. Experiments were repeated for reproducibility.

*Statistical Analysis:* Data analysis and statistical significance was determined using GraphPad Prism 7.01 software. Significance between the drug treated and control groups was determined by Turkey’s multiple comparison test with alpha set at 0.05. A P value of ≤ 0.05 was considered significant.

## Results

**Figure 1.**
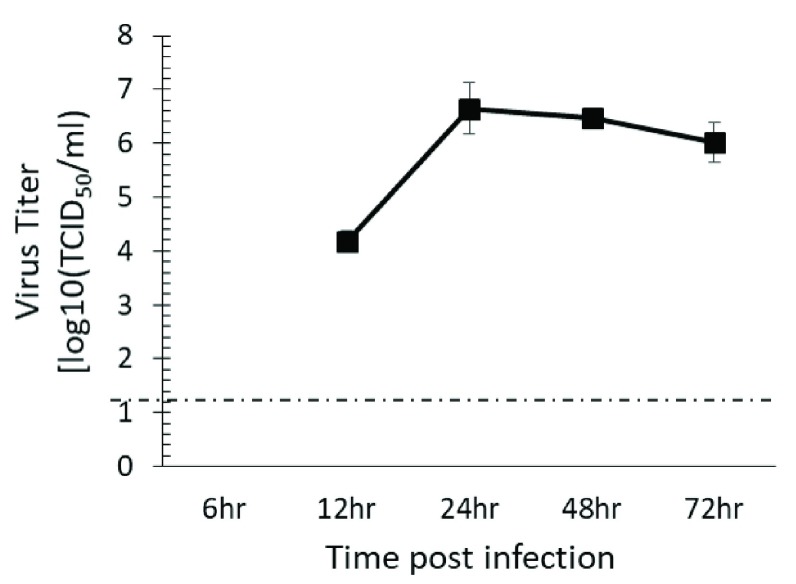
CHIKV 181/25 replication in hPDF cells. HPDF cells were infected with CHIKV 181/25 and virus titers were measured in the cell supernatants at 24 h post infection. Values presented as ± SEM. The dashed line indicates the limit of detection of assay.

*HPDF cells support CHIKV replication:* Dermal fibroblast are the target of CHIKV infection ^16^, therefore, replication kinetics of CHIKV181/25 was determined in hPDF. CHIKV181/25 was readily detected in the cell supernatants of hPDF cells, and reached peak titer at 24 h post infection ( Figure 1). Since 24 h post infection showed peak CHIKV181/25 titers in cell supernatants, this time point was chosen to test the effect of inhibitors on virus replication.

**Figure 2.**
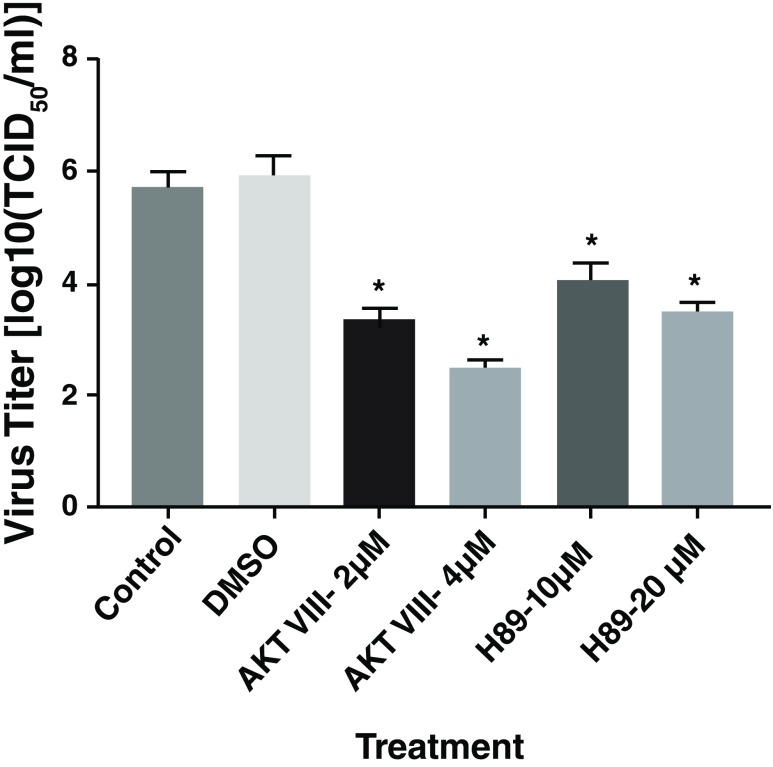
Effect of Pi3-akt signaling inhibitors on CHIKV replication. HPDF cells were pre-treated with inhibitors for 4–6 h and infected with CHIKV181/25. Significant reduction in virus titer was observed in the cells treated with AKT-VIII and H89.

*Akt-activation inhibitors inhibited CHIKV replication:* Effect of inhibition of Pi3-kinase and mTOR activation on CHIKV replication was tested by pre-treating hPDF cells with LY294002 and rapamycin, respectively. Both LY294002 and rapamycin did not affect CHIKV replication ( Supplementary figure 1A and B). Effects of inhibition of PKA and Akt-activation on CHIKV replication was tested by pre-treating hPDF cells with H89 and Akt-VIII, respectively. Both H89 and AKT-VIII significantly inhibited the CHIKV replication in hPDF cells ( Figure 2). Another Akt-activation inhibitor AKT-IV, which functions separately from AKT-VIII, also inhibited CHIKV replication ( Supplementary figure 2).

**Figure 3.**
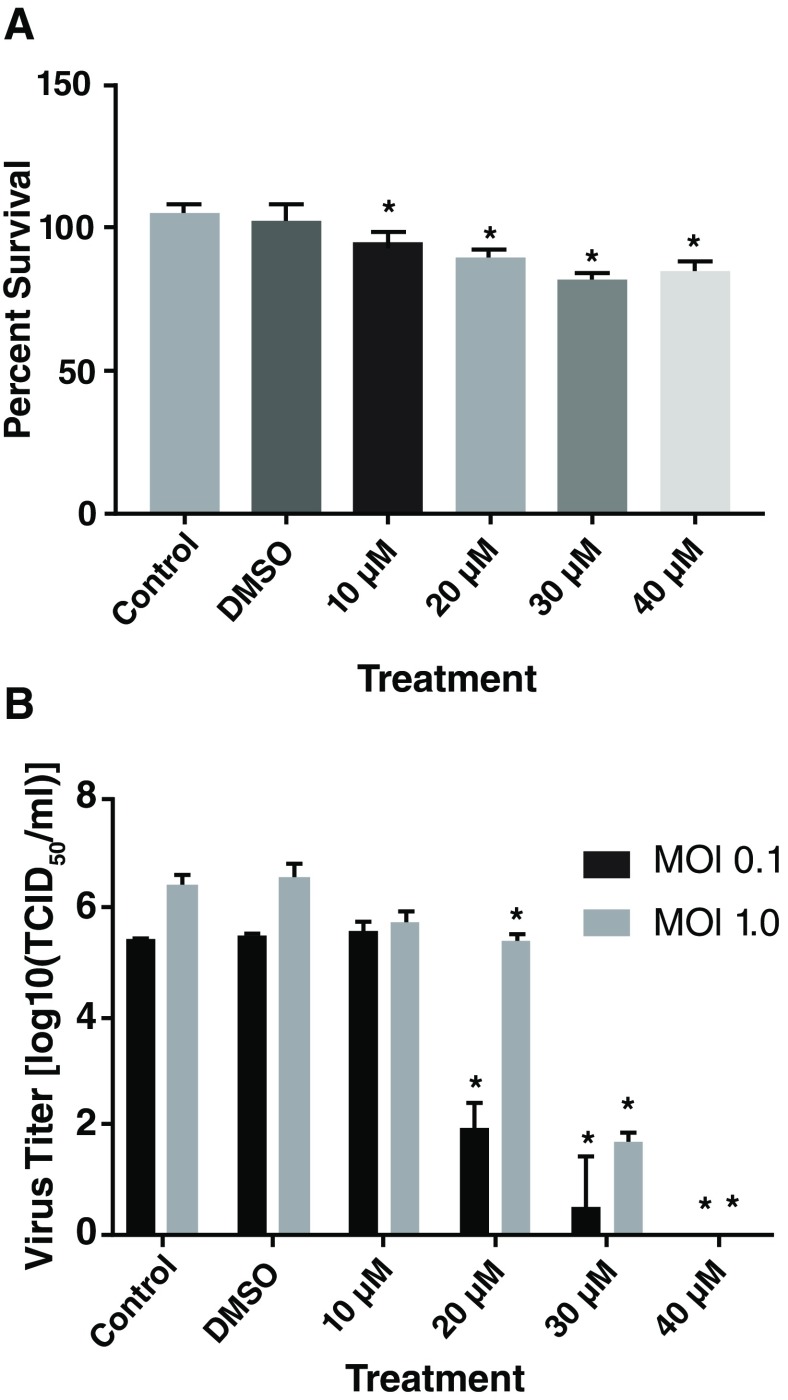
Effect of MF on hPDF cell proliferation and CHIKV181/25 replication. ( **A**) hPDF cells were treated with increasing doses of MF and MTT assay was done to determine the proliferation of cells. Since, MF was dissolved in dimethyl sulfoxide (DMSO), a DMSO concentration equivalent to concentration in 40µM dose was used in the assay. The control was saline treated cells. Significant effects on cell proliferation was observed in MF treated group compared to saline treated control groups. ( **B**) hPDF Cells were treated 4–6 h with MF and infected with CHIKV181/25 with either MOI 0.1 or 1. Virus titer in the cell supernatants was measured at 24 h post infection. Turkey’s multiple comparison test using 2 way ANOVA was performed to determine significance (*P < 0.05). Values are presented as ± SD. Values are presented as ± SD. * P ≤ 0.05 vs control group ( **A** & **B**), and ^ P <0.05 between two MOIs ( **B**). Data is representative of two repeat experiments.

**Figure 4.**
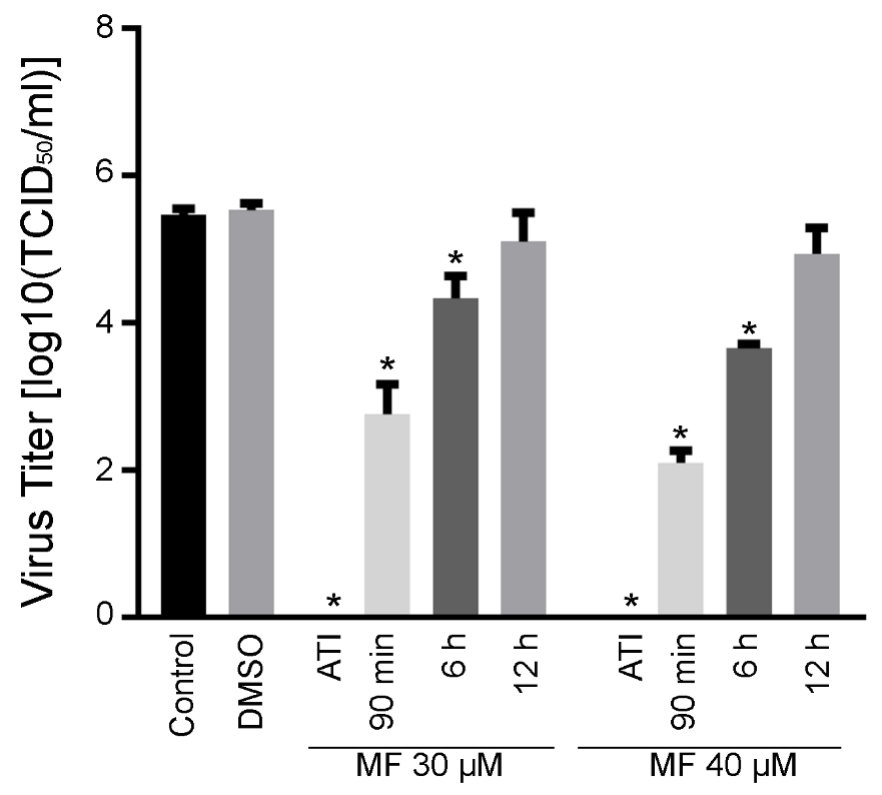
Effect of MF treatment post-infection on CHIKV replication. hPDF cells were infected with CHIKV181/25 (MOI = 1) and then treated with 30 or 40 µM MF. Virus titers were determined in the cell supernatants at 24 h post MF treatment. A significant reduction in virus titer was observed until 6 h pi. at the time of infection (ATI). Turkey’s multiple comparison using 2 way ANOVA test was performed to determine significance. Values are presented as ± SD. * P ≤ 0.05 as compared to respective saline control group. ^ P < 0.05 between the groups indicated on graph. Data is representative of two repeat experiments.

*Miltefosine inhibited CHIKV replication:* MF is a mitotic inhibitor, due to its Akt-phosphorylation inhibition activity, and MF treatment slightly reduced hPDF cell proliferation in comparison to saline or DMSO treated controls ( Figure 3A). Pre-treatment of cells with MF significantly inhibited CHIKV replication. Treatment with 20 µM and above doses of MF resulted in significant reduction in CHIKV titer in cell supernatants, however, at MOI=1, virus titers were significantly higher than the samples infected with MOI=0.1 ( Figure 3B). Therefore, extent of inhibition of CHIKV replication was dependent on the initial infectious load of the virus. To test the therapeutic potential of MF, hPDF cells were infected with CHIKV181/25 at MOI=1 and then treated with 30 or 40 µM dose of MF, either at the time of infection, 90 min, 6, or 12 h post-infection (pi). A significant inhibition of CHIKV replication was observed in samples treated with MF until 6 h pi ( Figure 4). Similar experiment with an MOI=0.1 showed inhibition of CHIKV replication until 12 h pi ( Supplementary figure 3).

**Figure 5.**
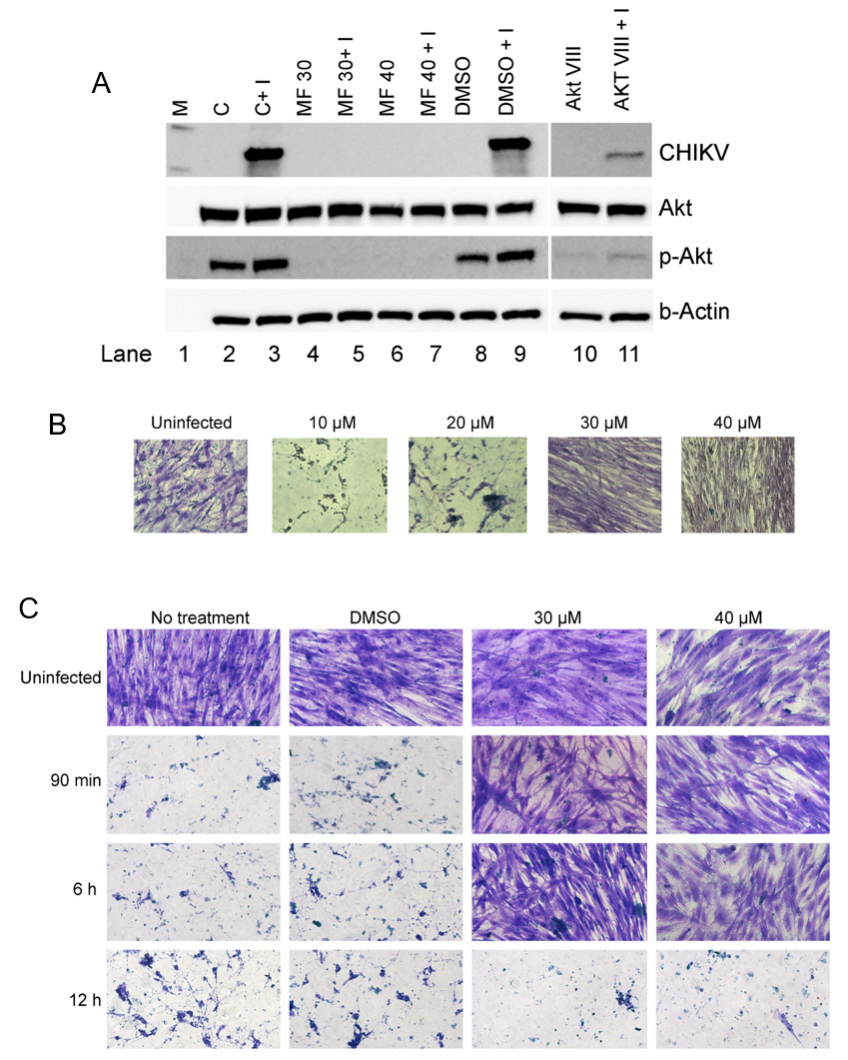
Effect of MF treatment on Akt-phosphorylation and cell growth in CHIKV infected cells. ( **A**) The cell lysates of hPDF cells infected with CHIKV181/25 were analyzed for native Akt, p-Akt, and CHIKV antigen levels by western blot analysis. Reduction in levels of CHIKV antigen was found to be concomitant with that of the reduction in the levels of p-Akt. M= ladder, C= Control uninfected cells, I = infected, and DMSO = dimethyl sulfoxide. ( **B**) Cells were treated with corresponding doses of MF for 4–5 h and infected with CHIKV181/25 (MOI=1). After 72 h pi, virus and drug were removed and cells were washed, replenished with fresh media and incubated for 4 days. ( **C**) Cells were infected with CHIKV181/25 (MOI=1) and treated with MF at the indicated time points post-infection. After 72 h pi, virus and drug were removed and cells were washed and replenished with fresh medium and incubated for 4 days. **B** and **C**: Cells were fixed and stained overnight with 10% neutral buffered formalin containing 0.1% crystal violet and subsequently washed gently under running tap water and observed under the microscope. Data is representative of two repeat experiments.

To check if reduction in CHIKV181/25 replication was associated with inhibition of Akt-phosphorylation, a western blot was performed on the hPDF cell-lysates collected from AKT-VIII or MF treated and CHIKV181/25-infected hPDF cells. CHIKV181/25 infection increased phosphorylated-Akt (p-Akt) levels in the cells over that of the uninfected controls ( Figure 5A: lanes 2 and 3). As expected, treatment with Akt-VIII reduced p-Akt levels in uninfected as well as CHIKV181/25-infected cells as compared to the respective controls, and this reduction was associated with reduction in CHIKV antigen levels ( Figure 5A: lanes 10, 11). Treatment with MF diminished the p-Akt levels both in the uninfected, as well as CHIKV181/25-infected cells, and was associated with diminished CHIKV antigen levels below the detection limit of the assay ( Figure 5A: lanes 4–7).

To determine if the inhibition of CHIKV181/25 replication by MF was complete or transient, re-activation of CHIKV181/25 replication in infected hPDF cells after the removal of MF from cell culture media was evaluated. HPDF cells were infected with CHIKV (MOI=1) and treated with MF (pre- or post-infection as described in methods section) for 72 h followed by washing of cells and replenishment with fresh growth media without MF. After 4 days a cellular monolayers were observed in CHIKV181/25-infected cells pre-treated with 30 and 40 µM of MF ( Figure 5B). In CHIKV181/25 infected cells post-treated with MF, cell growth was observed in samples treated with 30 and 40 µM of MF until 6 h pi (MOI =1) ( Figure 5C) and 12 h pi (MOI =0.1) ( Supplementary figure 4). These results suggest that MF-induced inhibition of CHIKV181/25 was complete at ≥30µM dose of MF.

To determine if the antiviral effects of MF was specific against CHIKV, effect of MF treatment on VEEV, TC83 strain, replication was evaluated. Unlike CHIKV181/25, no inhibition of TC83 replication (MOI=0.1) was observed by pretreatment of cells with low doses of MF, and reduction in virus titer was observed only with a 40µM dose of MF. Post-infection treatment of hPDF cells with MF did not affect TC-83 replication ( Supplementary figure 5), suggesting MF mediated inhibition to be more specific to CHIKV.

Raw data underlying the results presented Click here for additional data file. Copyright: © 2018 Sharma A et al. 2018 Data associated with the article are available under the terms of the Creative Commons Zero "No rights reserved" data waiver (CC0 1.0 Public domain dedication).

## Conclusion

CHIKV is a reemerging virus of global public health importance. Though not lethal, CHIKV causes a debilitating arthritic disease that severely affects the quality of life ^3, 4^. In the absence of specific therapeutic drugs or a vaccine, control of the CHIKV epidemic has been difficult and millions of people worldwide have been infected. Pi3-akt signaling has been shown to play an important role in viral infections. Akt-activation has been shown to be an important step during the infection of several viruses such as influenza, enterovirus and varicella zoster virus, and is suggested to help virus replication cycle by delaying or inhibiting apoptosis in the cells ^20– 23^. Phosphorylation of Akt and Akt-mediated Hsp90 activation has been shown during CHIKV replication ^12, 13, 15^. Pi3-akt signaling pathways was also found to be upregulated in white blood cells isolated from the CHIKV infected patients ^14^. Pi3-akt signaling pathway was also one of the top most pathways likely to be targeted by the modulated microRNAs following CHIKV infection ^11^. In this study, infection with CHIKV181/25 increased p-Akt levels in hPDF cells, which is in agreement with previous observation with wild type CHIKV in BHK cells ^15^. HPDF cells were used in this study as dermal fibroblast are the target of CHIKV infection and would provide relevant data for inhibition of CHIKV replication by drugs of interest ^16^. Specific inhibition of Akt-phosphorylation, but not of Pi3-kinase or mTOR activation, inhibited CHIKV replication. No effect on CHIKV protein expression has been shown after treatment with specific inhibitor of Pi3-kinase activation ^15^. Akt-VIII is a pleckstrin homology (PH) domain-dependent inhibitor of Akt-activation. The PH domain of Akt translocate Akt to the membrane where it is subsequently phosphorylated. To rule out the possibility of inhibition of CHIKV replication due to interference in translocation of Akt to the membrane, effect of AKT-IV inhibitor on CHIKV181/25 replication was tested. AKT-IV, which inhibits phosphorylation of Akt by inhibiting an upstream kinase other than Pi3-kinase, also inhibited CHIKV replication. Protein kinase A (PKA) regulate Akt-activation independent of Pi3-kinase and has been shown to play important role in viral infections ^24– 30^. Treatment of hPDF cells with H89, an inhibitor of PKA, also inhibited CHIKV181/25 replication. This observation was similar to the inhibition of hepatitis C virus by H89 reported elsewhere ^28^. Taken together, these results suggested that phosphorylation of Akt is important for CHIKV replication.

Miltefosine (MF) is an Akt-phosphorylation inhibitor, which is approved by the FDA for treating Leishmania infections in humans ^17^. It belongs to an alkylphosphocholine drug family and is an efficient inhibitor of Akt-phosphorylation ^31^. Therefore, as expected some reduction in hPDF cell proliferation was observed after treatment with MF. MF has also been shown to inhibit herpes simplex virus by inhibiting Akt-phosphorylation ^32^. Significant inhibition of CHIKV181/25 replication was observed in hPDF in prophylactic, as well as therapeutic treatment regimen. The antiviral activity was observed at 20–40 µM doses of MF, which correspond to 8.1–16.3 µg/ml of MF. In clinics, MF is administered orally as 50–100 mg/kg doses, which achieve plasma concentration ranging from 24–70 µg/ml with half-life of ~7 days ^17^. It is not possible to directly correlate *in-vitro* dose with *in vivo* doses. However, based on median plasma concentration and long half-life, MF may show anti-CHIKV activity *in vivo*.

To our knowledge this is the first study to report anti-CHIKV activity of MF. The study is limited by the use of attenuated strain of CHIKV, instead of the circulating wild-type strain of virus, which requires biosafety level 3 (BSL-3) containment for handling. However, we present a strong case for further evaluation of MF as anti-CHIKV drug *in vivo*, and against wild-type CHIKV virus.

## Data availability

The data referenced by this article are under copyright with the following copyright statement: Copyright: © 2018 Sharma A et al.

Data associated with the article are available under the terms of the Creative Commons Zero "No rights reserved" data waiver (CC0 1.0 Public domain dedication). 

Dataset 1: Raw data underlying the results presented DOI, 10.5256/f1000research.13242.d189063 ^33^

## Disclaimer

The opinions expressed herein are that of the author(s), and are not necessarily representative of those of the Uniformed Services University of the Health Sciences (USUHS), the Department of Defense (DOD), the United States Army, Navy, Air Force, and Defense Threat Reduction Agency.

## Supplementary material

**Supplementary File 1:** Supplementary Figure 1– Supplementary Figure 5

**Supplementary Figure 1: Effect of LY294002 and Rapamycin on CHIKV181/25 replication.** hPDF cells were pretreated 4–6 h with the inhibitor and infected with CHIKV181/25 at an MOI = 0.1. Virus titer in cell supernatant were measured at 24 h post infection. No effect on CHIKV replication was observed in cells pre-treated with either LY294002 (pi3k inhibitor; **A**) or rapamycin (mTOR inhibitor; **B**). Data is representative of two repeat experiment. Values are presented as ± SD.

**Supplementary Figure 2: Akt-IV inhibited CHIKV181/25 replication.** hPDF cells were pretreated 4–6 h with Akt-IV inhibitor and infected with CHIKV181/25 at an MOI = 0.1. Virus titer in cell supernatants was measured at 24 h post infection. Turkey’s multiple comparison test using 2 way ANOVA was performed to determine significance. Values are presented as ± SD. * P ≤ 0.05 as compared to control group. ^ P ≤ 0.05 as compared to control saline treated group. Data is representative of a two repeat experiment.

**Supplementary Figure 3: Effect of MF treatment post-infection on CHIKV replication**. A time dependent inhibition of CHIKV replication was observed in hPDF cells infected with CHIKV (MOI=0.1) and then treated with 40 µM of MF. A significant inhibition was observed until 12 h post infection. Cells that were treated after 24 h post infection did not show reduction in virus replication in cell supernatants. No virus titer was detected in the cell supernatant in the group treated with MF at the time of infection (ATI). Turkey’s multiple comparison test using 2 way ANOVA was performed to determine significance. Values are presented as ± SD. * P ≤ 0.05 as compared to control group. ^ P < 0.05 between the groups represented on the graph.

**Supplementary Figure 4: Effect of MF treatment on cell growth after infection with CHIKV181/25 with MOI 0.1:** ( **A**) Cells were treated with MF for 4–5 h and infected with CHIKV 181/25. After 72 h post infection, virus and drug were removed and cells were washed and replenished with fresh media and incubated for 4 days. ( **a**) Uninfected control; ( **b**) infected control; ( **c**) infected + 10 µM MF; ( **d**) infected + 20 µM MF; ( **e**) infected + 30 µM MF; and ( **f**) infected + 40 µM MF. ( **B**) Cells were infected with CHIKV181/25 (MOI=0.1) and treated with MF at the time post infection as indicated (ATI= at the time of infection). After 72 h post infection, virus and drug were removed and cells were washed and replenished with fresh medium.

**Supplementary Figure 5: TC83 replication in hPDF cells treated with Akt-activation inhibitor or MF**: ( **A**) HPDF cells were pretreated with MF for 4–6 h and infected with TC-83 at an MOI=0.1. ( **B**) HPDF cells were infected with TC-83 (MOI=0.1) and treated with MF 90 min after the infection. Values are presented as ± SEM. * P ≤ 0.05 as compared to DMSO treated group.

**Supplementary File 2:** Raw data underlying supplementary figures
