# Supplementary material for: Miltefosine inhibits Chikungunya virus replication in human primary dermal fibroblasts
Source: F1000Res. 2018 Jan 4;7:9. [Version 1] doi: 10.12688/f1000research.13242.1 (PMC5795271; doi:10.12688/f1000research.13242.1)
Supplement: Supplementary file 2 [file f1000research-7-14366-s0001.tgz › 2bb36364-ec90-4370-936b-8a63509c89a8.pdf]

Supplementary File 1: Supplementary Figures 1-5

Supplementary Figure 1:

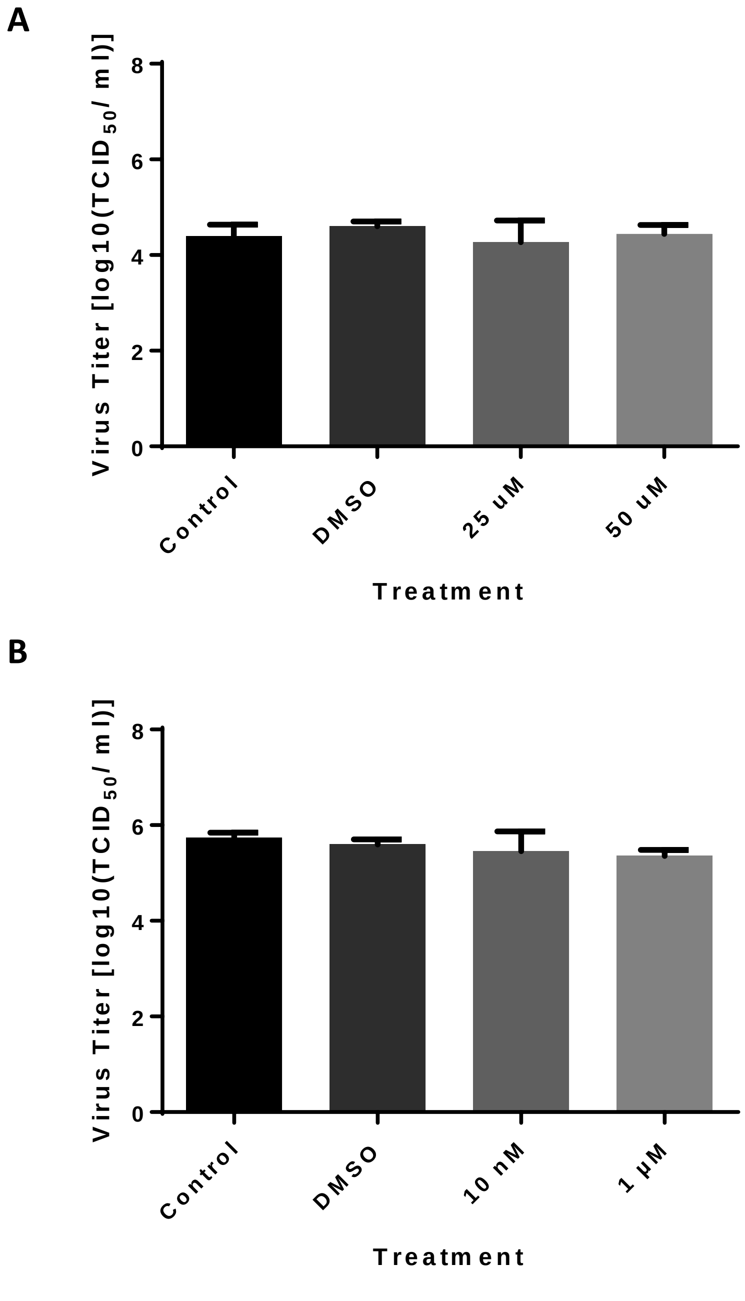

Effect of LY294002 and Rapamycin on CHIKV181/25 replication. hPDF cells were pretreated 4-6 h with the inhibitor and infected with CHIKV181/25 at an MOI = 0.1. Virus titer in cell supernatant were measured at 24 h post infection. No effect on CHIKV replication was observed in cells pre-treated with either LY294002 (pi3k inhibitor; **A**) or rapamycin (mTOR inhibitor; **B**). Data is representative of two repeat experiment. Values are presented as ± SD.

Supplementary Figure 2:

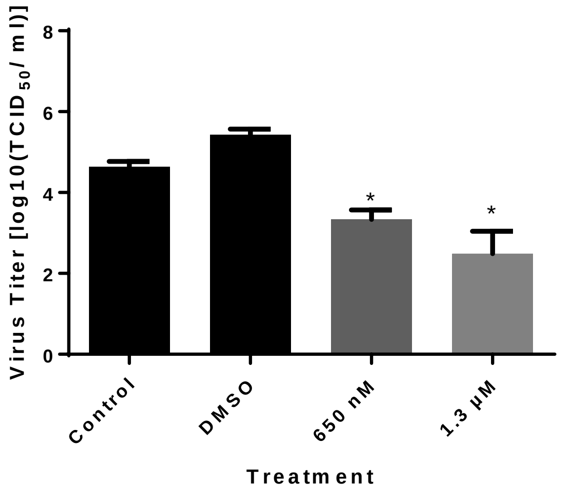

Akt-IV inhibited CHIKV181/25 replication. hPDF cells were pretreated 4-6 h with Akt-IV inhibitor and infected with CHIKV181/25 at an MOI = 0.1. Virus titer in cell supernatants were measured at 24 h post infection. Turkey's multiple comparison test using 2 way ANOVA was performed to determine significance. Values are presented as ± SD. * P ≤ 0.05 as compared to control group. ^ P ≤ 0.05 as compared to control saline treated group. Data is representative of a two repeat experiment.

Supplementary Figure 3:

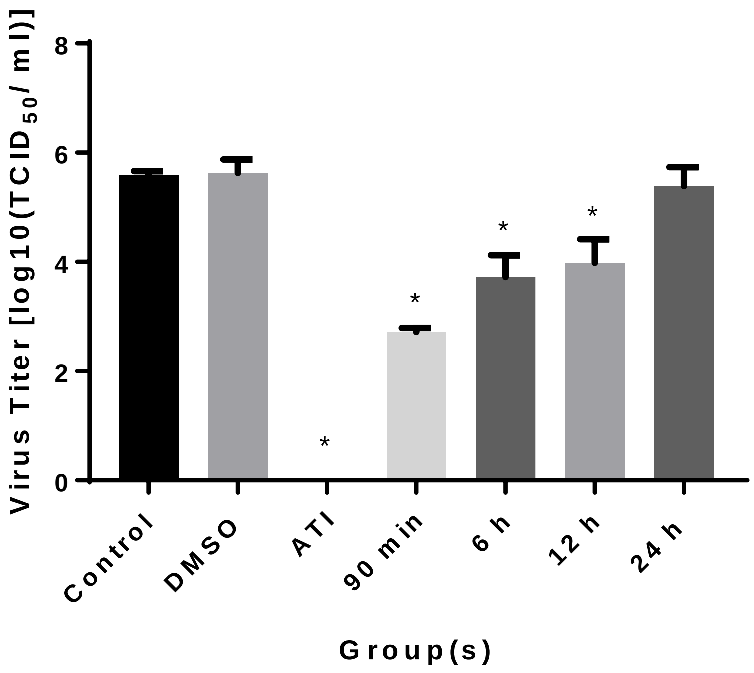

Effect of MF treatment post-infection on CHIKV replication. A time dependent inhibition of CHIKV replication was observed in hPDF cells infected with CHIKV (MOI=0.1) and then treated with 40 µM of MF. A significant inhibition was observed until 12 h post infection. Cells that were treated after 24 h post infection did not show reduction in virus replication in cell supernatants. No virus titer were detected in the cell supernatant in the group treated with MF at the time of infection (ATI). Turkey's multiple comparison test using 2 way ANOVA was performed to determine significance. Values are presented as ± SD. * $P \leq 0.05$ as compared to control group. ^ $P < 0.05$ between the groups represented on the graph.

Supplementary Figure 4:

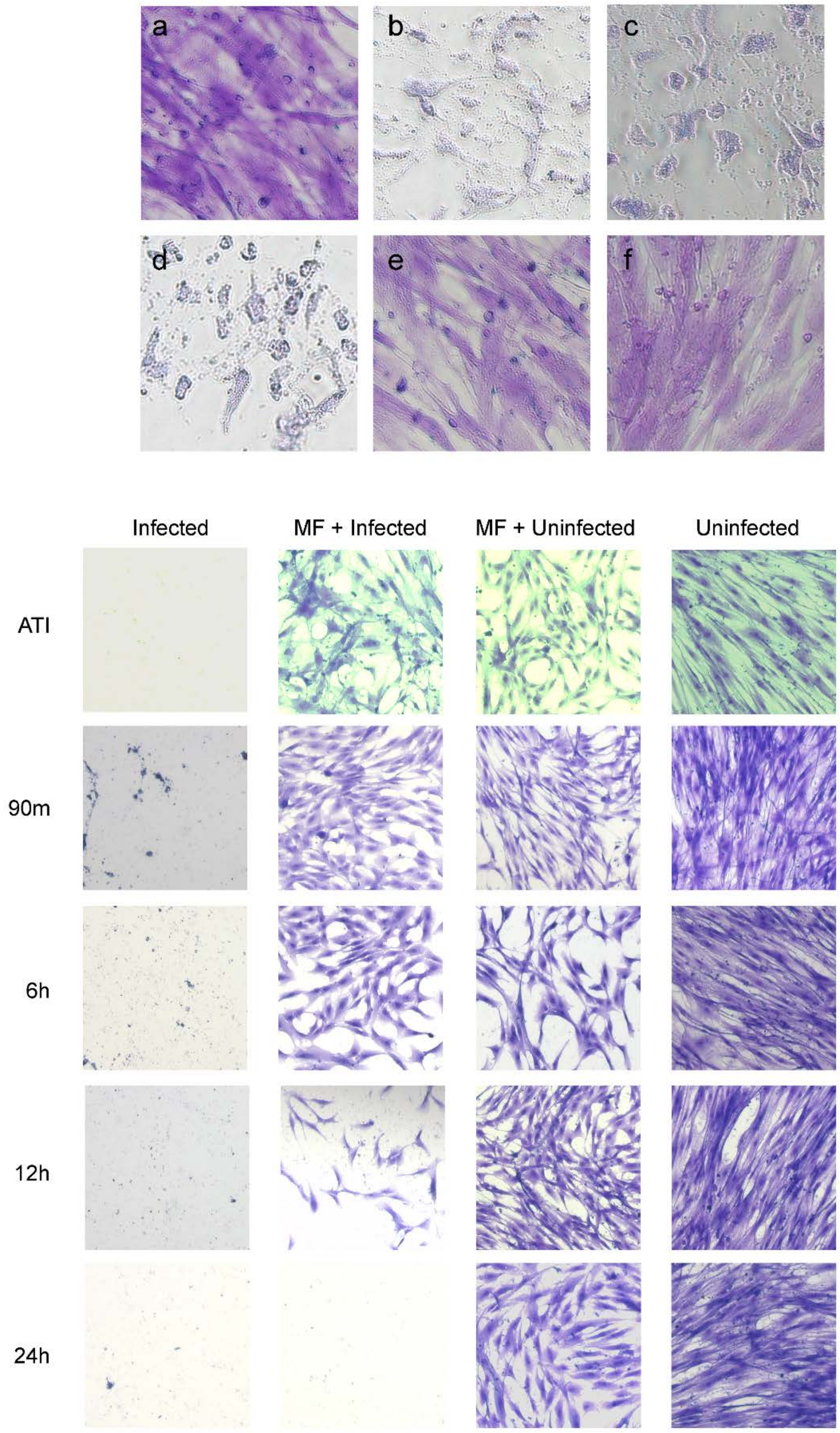

Effect of MF treatment on cell growth after infection with CHIKV181/25 with MOI 0.1: (**A**) Cells were treated with MF for 4-5 h and infected with CHIKV 181/25. After 72 h post infection, virus and drug were removed and cells were washed and replenished with fresh media and incubated for 4 days. (**a**) Uninfected control; (**b**) infected control; (**c**) infected + 10 µM MF; (**d**) infected + 20 µM MF; (**e**) infected + 30 µM MF; and (**f**) infected + 40 µM MF. (**B**) Cells were infected with CHIKV181/25 (MOI=0.1) and treated with MF at the time post infection as indicated (ATI= at the time of infection). After 72 h post infection, virus and drug were removed and cells were washed and replenished with fresh medium.

Supplementary Figure 5:

A

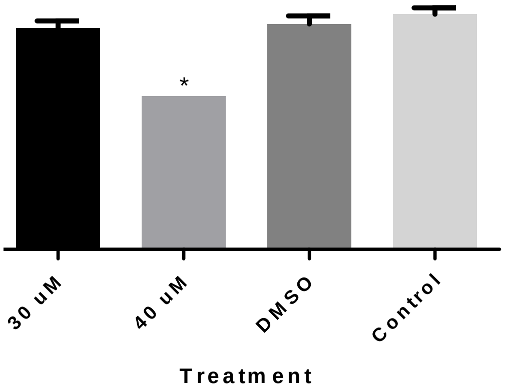

B

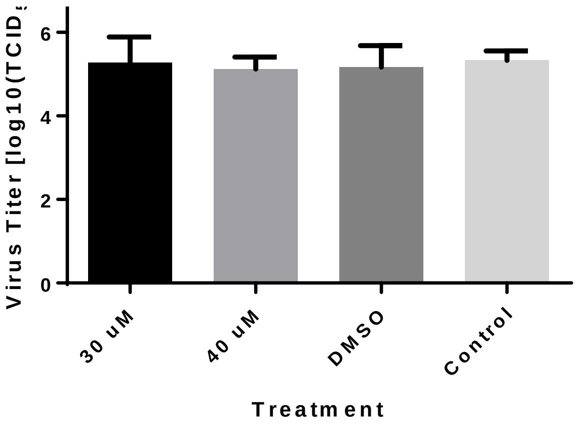

TC83 replication in hPDF cells treated with Akt-activation inhibitor or MF: (**A**) HPDF cells were pretreated with MF for 4-6 h and infected with TC-83 at an MOI=0.1. (B) HPDF cells were infected with TC-83 (MOI=0.1) and treated with MF 90 min after the infection. Values are presented as ± SEM. * $P \leq 0.05$ as compared to DMSO treated group.
