# Supplementary material for: Miltefosine inhibits Chikungunya virus replication in human primary dermal fibroblasts
Source: F1000Res. 2018 Jan 4;7:9. [Version 1] doi: 10.12688/f1000research.13242.1 (PMC5795271; doi:10.12688/f1000research.13242.1)
Supplement: Peer review history

# Peer review: Referee response for version 1

**DOI:** 10.5256/f1000research.14366.r29844

**Published:** 2018-02-01

**Authors:** Rana Abdelnabi

**Affiliations:** 1 Department of Microbiology and Immunology, KU Leuven - University of Leuven, Leuven, Belgium

The manuscript is well written and the methodology is clear. However I have only minor comments:

1. Pi3-akt signaling should be briefly introduced in the Introduction section.
2. The toxicity data of the used inhibitors should be included in the supplementary material.
3. Is there any explanation why the virus controls in supplementary Figure 1B and supplementary Figure 2 are almost 1 log less than the virus controls in the rest of figures although MOI=0.1 was used in all these experiments?.

I have read this submission. I believe that I have an appropriate level of expertise to confirm that it is of an acceptable scientific standard.

# Peer review: Referee response for version 1

**DOI:** 10.5256/f1000research.14366.r29502

**Published:** 2018-02-01

**Authors:** Soma Chattopadhyay

**Affiliations:** 1 Institute of Life Sciences, Bhubaneswar, India

In the manuscript by Sharma et al., entitled “ **Miltefosine inhibits Chikungunya virus replication in human primary dermal fibroblasts**”, the authors have shown that MF, an FDA-approved

 Akt-inhibitor, inhibited CHIKV replication in pre- and post-infection treatment regimens And proposed that  Akt-phosphorylation can be an amenable target of therapy against CHIKV infection.

 The work is interesting and written properly. However, few things have to be corrected before consideration.

**Major comments**:

1. What is the % of inhibition / reduction, that has been never been mentioned in case of all the inhibitors.
2. How all the statistical values are with one *?
  Fig 3 A- How 10 μM is statistically significant?
  Fig 3 B- How all the bar diagrams are with same p-value, actual p value should be provided here.
3. How the reduction of viral titer is shown at 24 hpi in Fig 3, where as in Fig 4 the significant reduction is shown only at 6 hpi and then the infection rate has been decreased.
4. How the current manuscript has been used as reference in this manuscript. The data is the part of this manuscript only. This cannot be referred.
5. What is the need for showing saline treated cells as control has not been explained in the text.
6. What are the specifications of all the inhibitors has not been explained. Why LY294002 and Rapamycin did not work, can the author give some explanation?
7. Little more introductions on different drugs inhibiting CHIKV replication may be mentioned in one para in the introduction.

I have read this submission. I believe that I have an appropriate level of expertise to confirm that it is of an acceptable scientific standard, however I have significant reservations, as outlined above.

# Peer review: Referee response for version 1

**DOI:** 10.5256/f1000research.14366.r29497

**Published:** 2018-01-19

**Authors:** Mukesh Kumar

**Affiliations:** 1 Department of Tropical Medicine, Medical Microbiology and Pharmacology, University of Hawaii at Manoa, Honolulu, HI, USA

The manuscript entitled " Miltefosine inhibits Chikungunya virus replication in human primary dermal fibroblasts” by Sharma and colleagues evaluated the effect of AKT-activation inhibitors on Chikungunya virus replication. The manuscript is well written and easily comprehensible. The results of this work are very significant to the field and fit with the scope of this journal. The manuscript would be improved by consideration of the points below, and amendment as indicated:

 Similar to MF, percent cell survival after treatment with AKT-VIII and H89 should be included.

 Y-axis missing in Supp. Fig. 5A

I have read this submission. I believe that I have an appropriate level of expertise to confirm that it is of an acceptable scientific standard.
